# High‐Throughput Generation of Tumor Spheroids via Droplet Microfluidics for siRNA‐Loaded Nanomedicine Assessment

**DOI:** 10.1002/adhm.202503604

**Published:** 2026-02-21

**Authors:** Ling Liu, Guoying Wang, Yang Zhang, Bingyang Shi, Ming Li

**Affiliations:** ^1^ School of Engineering Macquarie University Sydney New South Wales Australia; ^2^ School of Mechanical and Manufacturing Engineering The University of New South Wales Sydney New South Wales Australia; ^3^ Macquarie Medical School Faculty of Medicine, Health and Human Sciences Macquarie University Sydney New South Wales Australia; ^4^ School of Biomedical Engineering The University of Technology Sydney Sydney New South Wales Australia

**Keywords:** droplet microfluidics, high‐throughput generation, nanomedicine, tumor spheroids

## Abstract

Tumor spheroids, the most widely used model of 3D cell culture, have emerged as a viable platform for assessing drug responses. However, high‐throughput validation of novel drugs using tumor spheroids remains hindered by the challenges in generating large‐scale, homogeneous, and functionally relevant spheroids. Here, a flow‐focusing droplet microfluidic platform is developed for high‐throughput generation of uniform tumor spheroids, producing over 50 000 droplets within 5 min, with each microdroplet serving as an individual bioreactor for spheroid formation. The initial size of the tumor spheroids is tuned based on cell concentration and water‐to‐oil flow rate ratio during microdroplet generation. After being released from the microdroplets, the 3D tumor spheroids continue growing, reaching diameters exceeding 300 µm. The growth and functional characteristics of the spheroids are examined both in a liquid environment and in a 3D collagen matrix. Moreover, these tumor spheroids enable assessment of the therapeutic efficacy of siRNA‐based nanomedicine that demonstrates enhanced performance compared to free siRNA treatments. This platform offers a robust and scalable approach for evaluating novel nanomedicines, providing valuable insights into their therapeutic potential and underlying mechanisms of action.

## Graphical abstract

A droplet microfluidic platform is employed to enable high‐throughput, uniform tumor spheroid generation for evaluating siRNA‐loaded nanomedicines at the protein level. As a proof of concept, breast (MCF‐7) and brain (U87 MG) cancer cell lines are investigated using this platform, revealing penetration profiles and therapeutic responses between the two spheroid models.

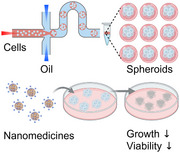

## 1 Introduction

Tumor spheroids, formed through self‐assembly of spherical cell aggregates, have attracted great attention due to their well‐defined 3D structure and their ability to closely mimic in vivo tumor characteristics [1, 2]. Within these 3D structures, cells engage in extensive intercellular interactions and establish physiologically relevant gradients of nutrients, oxygen, and signaling molecules, thereby effectively recapitulating critical aspects of the tumor microenvironment [1, 3]. This biomimetic nature enables tumor spheroids to serve as a valuable model for investigating cancer pathophysiology and evaluating the efficacy of anticancer therapeutics [4, 5]. Compared to conventional 2D cell cultures, tumor spheroids exhibit remarkable differences in drug responses, including variations in drug sensitivity, resistance, and penetration depths [6, 7, 8]. These factors are essential for improving the reliability and translational relevance of preclinical drug discovery and development. Furthermore, the use of tumor spheroids can reduce the reliance on animal models [9], addressing ethical concerns and streamlining research timelines. These advantages highlight the important role of tumor spheroids in advancing cancer biology and therapeutic development.

A variety of techniques, such as hanging drop method [10], liquid overlay culture [11], and rotating wall vessel systems [12], have been employed to generate tumor spheroids. However, these conventional methods often fall short of meeting the requirements for high‐throughput drug screening due to their inherent limitations, including low throughput, labor‐intensive procedures, and large size variability [13]. The generation of tightly packed and size‐homogeneous spheroids with high reproducibility, scalability, and compatibility with long‐term culture remains a major bottleneck [5, 14]. Microfluidic technologies offer a promising solution to overcome these limitations, enabling precise control over fluid flow and local microenvironment to facilitate scalable spheroid production. Among these, droplet microfluidics has emerged as a particularly effective platform, surpassing microwell, microstructure, and field‐driven microfluidic‐based platforms in throughput and control [15]. Their ability to encapsulate multiple cells within discrete microdroplets allows for the generation of highly uniform spheroids, while also offering flexibility in tuning microenvironmental conditions to better mimic in vivo tumor biology [16, 17].

Different formats of droplet‐based microfluidic platforms, such as liquid droplets [18, 19], hydrogel microdroplets [20, 21], and core–shell microcapsules [22] have been successfully employed to generate tumor spheroids with high uniformity and throughput. Moreover, tumor spheroids generated via droplet microfluidic platforms have been utilized in diverse therapeutic evaluations, including traditional chemotherapeutic agents (e.g., doxorubicin) [19, 23], photothermal interventions [24], and cell‐based therapies [25, 26]. However, despite the demonstrated efficiency and adaptability of droplet microfluidics in producing large numbers of spheroids, their application in assessing the therapeutic efficacy of nanomedicines remains relatively limited and underexplored [17]. In particular, the use of droplet‐based spheroid systems for systematic and high‐throughput functional evaluation of nanomedicines, especially using quantitative protein‐level readouts, has rarely been demonstrated. Expanding the use of these systems to nanomedicine research presents an important opportunity to accelerate cancer therapeutic drug development by providing scalable platforms for high‐throughput screening and mechanistic investigation.

Nanoparticle‐based therapeutics have recently gained significant clinical recognition [27], with several nanomedicines receiving approval from regulatory agencies including the U.S. Food and Drug Administration and the European Medicines Agency [28]. To accelerate the development of cancer nanomedicines, there is a critical need for preclinical screening platforms that utilize 3D tumor models [27]. While tumor spheroids have been increasingly adopted as in vitro tools for nanomedicine evaluation, many prior studies have used them primarily as simplified models, with limited emphasis on scalable, high‐throughput screening applications [29]. In this study, we introduce a droplet‐based microfluidic platform capable of generating highly uniform tumor spheroids for the evaluation of nanoparticle‐mediated drug delivery. The formation, release, and long‐term culture of tumor spheroids generated within this platform were investigated. As proof of concept, we assessed the therapeutic efficacy of gene‐silencing small interfering RNA (siRNA)‐loaded nanoparticles within 3D tumor spheroids. This work demonstrates the application of a droplet‐based spheroid system for high‐throughput, quantitative functional evaluation of siRNA nanomedicine at the protein level, addressing an underexplored aspect of nanomedicine assessment in 3D tumor models. To the best of our knowledge, systematic, high‐throughput, and protein‐level evaluation of siRNA nanomedicine using large‐scale microdroplet‐generated tumor spheroids has not been previously reported.

## 2 Results

**FIGURE 1:**
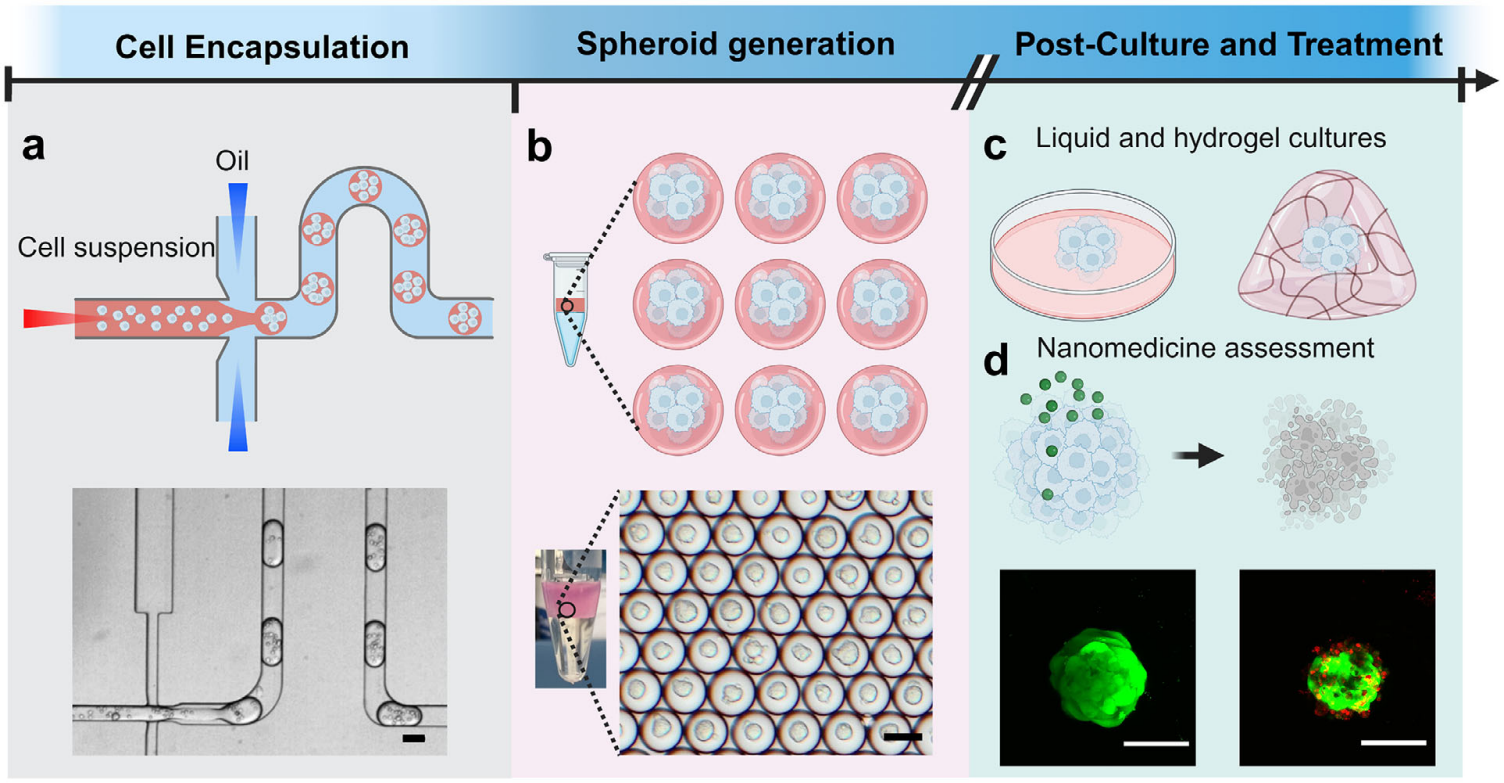
Overview of tumor spheroid generation using a droplet‐based microfluidic platform for nanomedicine evaluation. (a) Encapsulation of tumor cells within microdroplets at a flow‐focusing region of the microfluidic device. (b) Formation of tumor spheroids within microdroplets. (c) Culture of spheroids released from microdroplets in liquid medium and hydrogel matrix environments to support further growth and development. (d) Application of the developed tumor spheroids for therapeutic assessment of siRNA‐loaded nanomedicine. Scale bars: 100 µm.

This study aims to investigate the application of droplet microfluidics for the generation of tumor spheroids, enabling the high‐throughput evaluation of nanomedicine efficacy. A flow‐focusing droplet microfluidic device was employed to encapsulate tumor cells within uniform water‐in‐oil microdroplets (Figure 1a). Specifically, a cell suspension in culture medium served as the aqueous phase, which was sheared into discrete cell‐encapsulating microdroplets at the flow‐focusing junction by the continuous oil phase. These monodisperse liquid microdroplets act as isolated microenvironments that support controlled cellular aggregation and spheroid formation (Figure 1b). Following droplet incubation, the resulting spheroids are released and can be maintained either in standard liquid culture or embedded within a 3D hydrogel matrix to support further growth and development (Figure 1c). Well‐formed spheroids are subsequently used to evaluate the therapeutic performance of siRNA‐loaded nanomedicine, providing a reliable platform for nanomedicine assessment (Figure 1d).

### 2.1 Tumor Spheroid Generation

**FIGURE 2:**
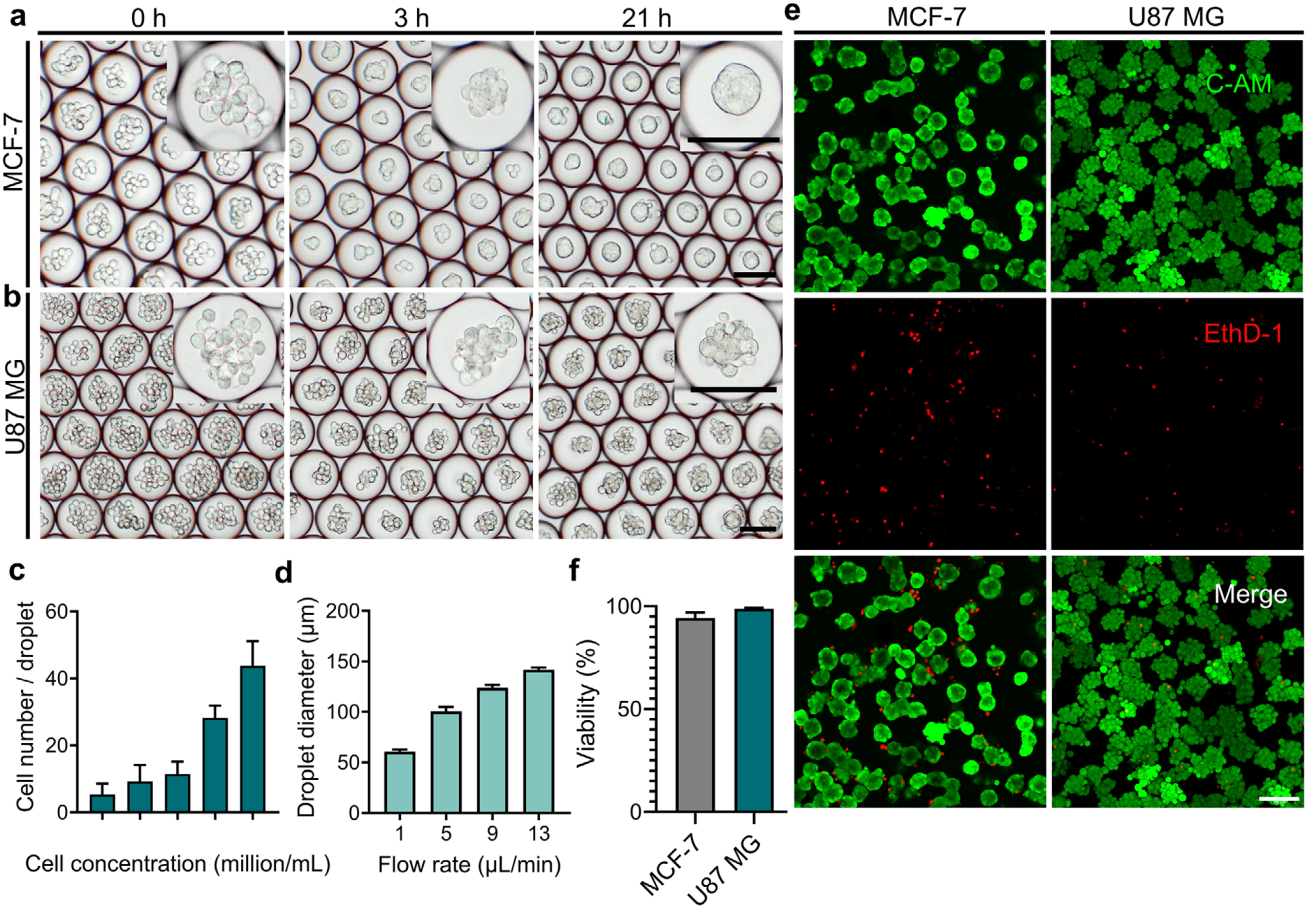
Formation and characterization of tumor spheroids generated via droplet microfluidics. (a) Time‐lapse images showing the morphological changes of MCF‐7 cells within droplets (0, 3, and 21 h). (b) Morphological changes of U87 MG cells within droplets (0, 3, and 21 h). (c) Relationship between initial cell concentration and the average number of cells encapsulated per droplet under fixed flow rate (water: oil = 9:15 µL/min, *n* = 50 for each condition). (d) Correlation between water flow rate and droplet diameter under constant oil flow conditions (15 µL/min, *n* = 50 for each condition). (e) Representative fluorescence images of Calcein‐AM/EthD‐1‐stained spheroids, indicating live (green) and dead (red) cells. (f) Quantitative analysis of cell viability in spheroids released from droplets (*n* = 3). Scale bars: 100 µm. Data are presented as mean ± SD.

The aggregation and spheroid formation of MCF‐7 and U87 MG cells within microdroplets at 0, 3, and 21 h are shown in Figure 2a,b. At the initial time point (0 h), individual cells were observed clustering near the center of the droplets, likely due to the curved interface of the droplets [30]. After 3 h of incubation, both cell lines showed noticeable aggregation. By 21 h, spheroid formation was observed in both cell types, with distinct morphological differences. MCF‐7 spheroids displayed compact, spherical structures with well‐defined, smooth boundaries and no visible individual cells. In contrast, U87 MG spheroids appeared less cohesive, with less regular outlines and loosely associated cells. These differences in spheroid morphology may be attributed to intrinsic cellular characteristics, particularly the non‐invasive phenotype and high expression of junctional proteins such as E‐cadherin in MCF‐7 cells, which promote tighter intercellular adhesion [31].

The spheroid size depended closely on the number of cells encapsulated in each droplet, which could be regulated by adjusting the initial cell density. Figure 2c presents the relationship between cell concentration and the average number of encapsulated cells in each droplet under fixed water‐to‐oil phase flow rates of 9:15 µL/min. Using cell concentrations ranging from 10 to 50 million cells/mL, the number of encapsulated cells per droplet varied from 5 ± 3.23 to 44 ± 7.33. Theoretically, a droplet with a diameter of 120 µm corresponds to a volume of approximately 0.90 nL, yielding an expected cell count between 9 and 45 per droplet. Experimental results were in reasonable agreement with the theoretical estimates, demonstrating the overall controllability and reproducibility of the system. At the highest concentration of 50 million cells/mL, variability in the number of encapsulated cells per droplet increased significantly. This was primarily due to the sedimentation of cells within the suspension over time, particularly at higher concentrations, which led to uneven cell distribution in the aqueous phase. Moreover, increased cell concentrations promoted cell‐cell contact and aggregation prior to droplet formation, further compromising the uniformity of cell encapsulation.

In addition to adjusting cell concentration, the water‐to‐oil flow rate ratio can influence droplet size, thereby modulating cell encapsulation. Figure 2d shows that, under a fixed oil phase flow rate of 15 µL/min, increasing the aqueous flow rate from 1 to 13 µL/min produced droplets with diameters ranging from approximately 61 ± 2.29 to 142 ± 2.21 µm. The system produces over 50 000 droplets every 5 min at a specific water‐to‐oil flow rate (9:15 µL/min), demonstrating high‐throughput capability. Stable droplet formation was maintained across this range and could be extended to higher oil‐phase flow rates, up to 50 µL/min (Figure S3). Moreover, spheroids generated by the droplet microfluidic platform exhibited a narrow size distribution within a single batch, with most spheroids clustered within a confined diameter range. When spheroid diameters from three independent batches were pooled, a compact and concentrated size distribution with limited dispersion was observed, indicating consistent size control by the platform (Figure S4).

These findings highlight the tunability and robustness of the microfluidic platform, particularly for applications requiring the generation of large numbers of spheroids. By increasing droplet generation frequency through higher flow rates, the total processing time can be significantly reduced, minimizing issues such as cell sedimentation and uneven distribution during encapsulation. This capability is especially important for high‐throughput applications, including drug screening.

### 2.2 Tumor Spheroid Characterization

**FIGURE 3:**
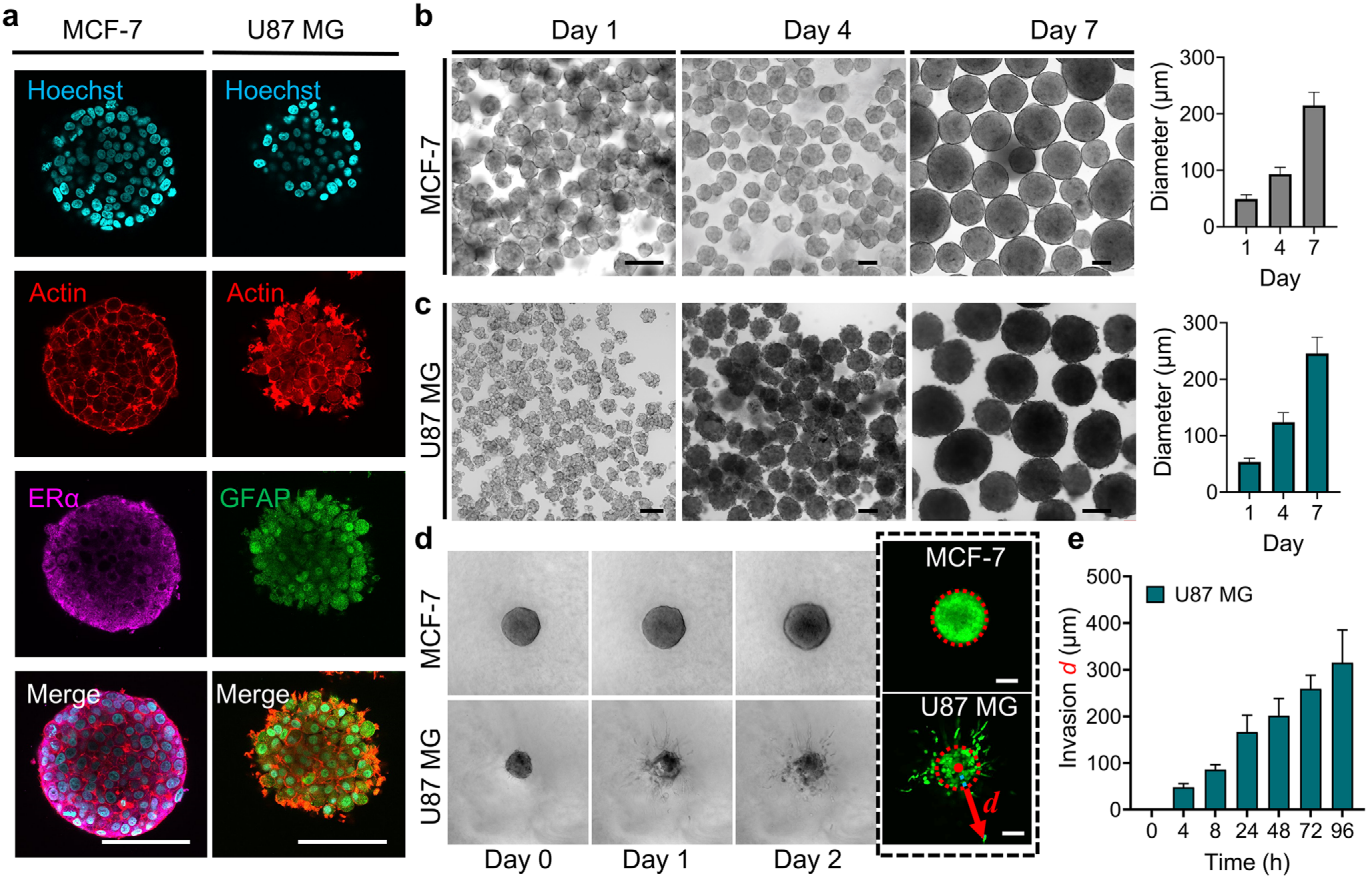
Characterization and growth of tumor spheroids following release from microdroplets. (a) Immunofluorescence staining of MCF‐7 and U87 MG tumor spheroids. (b,c) Morphological evolution and diameter increase of MCF‐7 and U87 MG spheroids over time (Days 1, 4, and 7). (*n* = 45 for each condition). (d) Representative microscopic images showing morphological changes of MCF‐7 spheroid and U87 MG spheroid within collagen type I on Day 0 (initial embedding), Day 1, and Day 2. (e) Time‐dependent invasion distance of U87 MG spheroids embedded in collagen type I (*n* = 3). Scale bars: 100 µm. All data are presented as mean ± SD.

Although the platform can provide rapid and controlled spheroid formation, the enclosed droplet environment restricted long‐term culture of tumor spheroids due to limited nutrient and oxygen exchange. Cell viability began to decline after extended culture in closed droplets, causing the disintegration of the spheroid culture [17]. Therefore, spheroids were released from the droplets into a suitable external environment to enable further culture or analysis. After release, cell viability was evaluated through staining with Calcein‐AM and EthD‐1, as shown in Figure 2e,f. Both MCF‐7 and U87 MG tumor spheroids exhibited high viability, averaging approximately 94.29% and 98.78%, respectively, with minimal presence of dead cells. Moreover, immunofluorescence staining was performed to confirm the cellular identity and tumor‐specific characteristics of the spheroids (Figure 3a). U87 MG spheroids expressed glial fibrillary acidic protein (GFAP), while MCF‐7 spheroids showed positive staining for estrogen receptor alpha (ERα). The cytoskeletal structure and nuclei of the spheroids were imaged using Actin and Hoechst staining, respectively, confirming the structural organization of the spheroids. These results confirm that this droplet microfluidic system for spheroid formation supports high cell viability and is compatible with subsequent long‐term culture and functional assays.

### 2.3 Tumor Spheroid Growth after Release

The growth characteristics of tumor spheroids released from microdroplets were systematically evaluated. Both MCF‐7 spheroids and U87 MG spheroids were released and subsequently cultured in either liquid suspension or a 3D hydrogel matrix. When maintained in liquid medium, both spheroid types exhibited significant growth over time, with their average diameters increasing from approximately 50 µm on Day 1 to over 200 µm after 7 days of culture (Figure 3b,c). It is important to note that spheroid density in suspension cultures must be carefully regulated to avoid unintended merging, which can compromise the uniformity and structural integrity of individual spheroids. This merging phenomenon occasionally led to the formation of abnormally large spheroids, with diameters exceeding 700 µm (Figure S5), which were excluded from the growth curve analysis.

To evaluate the invasive potential of the spheroids, both cell types were embedded in a 3D collagen I matrix. As shown in Figure 3d,e, U87 MG spheroids exhibited significant invasion. Cell infiltration into the surrounding collagen was observed as early as 4 h post‐embedding. The spheroid boundary was defined as the origin, and the furthest extent of cell invasion was measured as the invasion distance (*d)*. The average *d* of U87 MG spheroids reached 315 µm after being cultured for 4 days in gel. In contrast, MCF‐7 spheroids exhibited no significant invasive activity. They showed an increase in overall spheroid size, similar to what was observed in liquid culture conditions. This is likely due to their non‐invasive phenotype and high E‐cadherin expression [31]. These findings highlight the distinct invasive behaviors of the two tumor spheroid models and further validate their utility in modeling tumor‐specific phenotypes.

### 2.4 Evaluation of Nanoparticle Penetration into Tumor Spheroids

**FIGURE 4:**
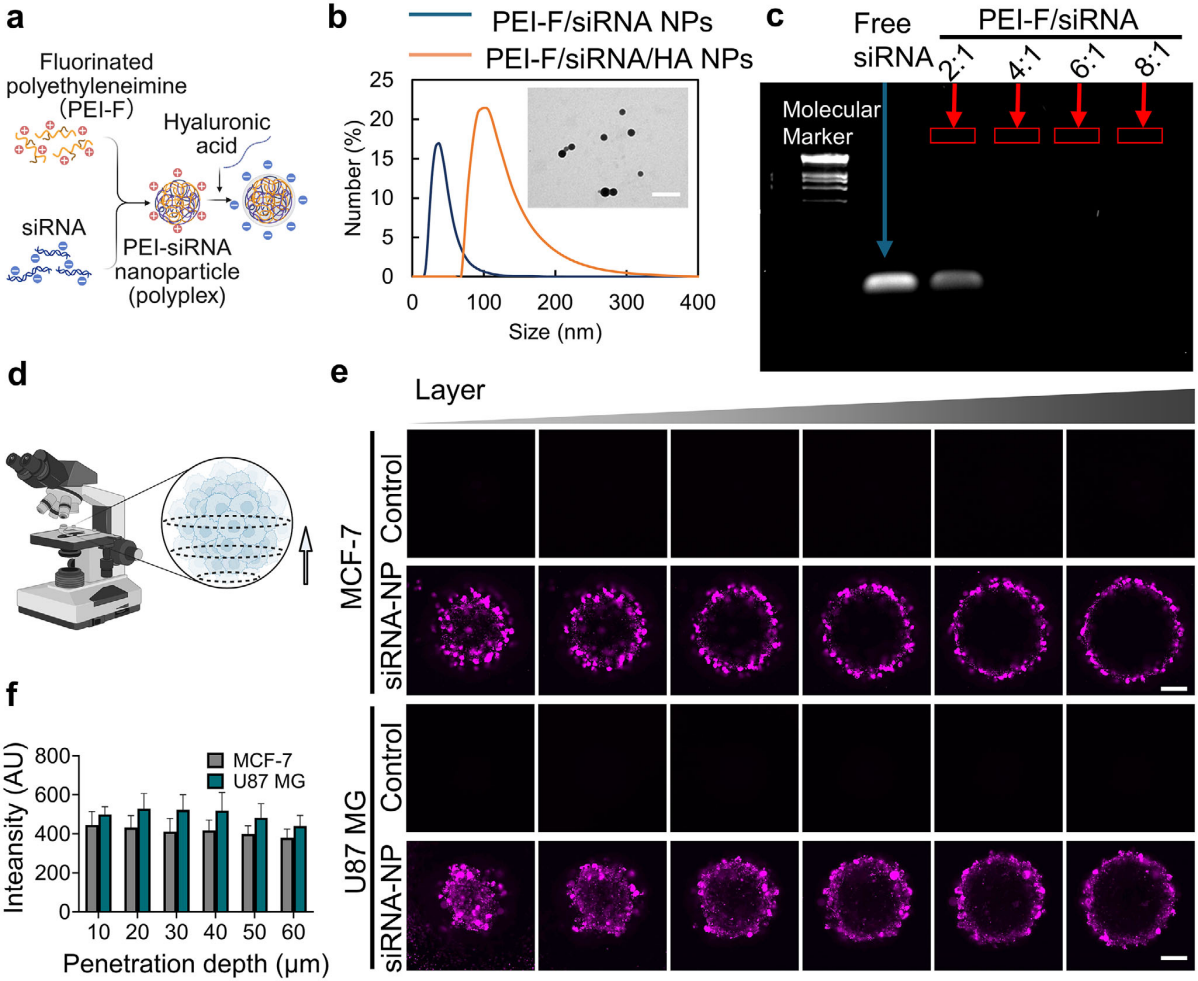
Evaluation of nanoparticle penetration into 3D tumor spheroids. (a) Schematic illustration of PEI‐F/siRNA/HA NPs. (b) Size distribution of PEI‐F/siRNA NPs and PEI‐F/siRNA/HA NPs, and TEM image (inset) of PEI‐F/siRNA/HA NPs. Scale bar: 500 nm. (c) Gel electrophoresis results of siRNA encapsulation efficiency at various mass ratios between PEI‐F and siRNA. (d) Schematic illustration of confocal imaging of tumor spheroids, scanned from bottom to center. (e) Representative Z‐stack confocal fluorescence images showing Cy5‐labeled siRNA nanoparticle penetration from the bottom to the center of the spheroids. Scale bars: 100 µm. (f) Quantitation of Cy5 fluorescence intensity within MCF‐7 and U87 MG spheroids after 6 h incubation. *n* = 3 biological replicates. Data are presented as mean ± SD. Control: spheroids treated with free siRNA‐Cy5; siRNA‐NP: spheroids treated with PEI‐F/siRNA‐Cy5/HA NPs.

To further assess the applicability of microdroplet‐generated tumor spheroids for nanomedicine evaluation, small interfering RNA (siRNA)‐based nanoparticles were employed as a proof of concept. Recently, siRNA therapy has attracted increasing attention as an effective cancer therapeutic tool due to its ability to selectively silence oncogenes at the post‐transcriptional level [32, 33]. However, challenges such as siRNA instability and poor cellular uptake hinder its clinical translation. Nanoparticle (NP) carriers address these limitations by protecting siRNA from degradation and facilitating its efficient delivery to target cells [34]. Here, fluorinated low‐molecular‐weight polyethyleneimine (1.8 K) (PEI‐F) was used as the siRNA carrier, with a hyaluronic acid (HA) coating to target the CD44 receptor (Figure 4a), which is commonly overexpressed in various tumor cells [35, 36]. Following siRNA loading, the PEI‐F/siRNA complex exhibited an average hydrodynamic diameter of 40.3±8.6 nm (Figure 4b) and a surface charge of +30 mV. After coating with HA, the NP diameter increased to about 117.6±1.3 nm, and the surface became negatively charged, measuring −30 mV, indicating successful surface modification. The synthesized nanoparticles exhibited a uniform size distribution, as confirmed by transmission electron microscopy (TEM). Gel electrophoresis analysis demonstrated that when the weight ratio of PEI‐F to siRNA was above 4:1, the nanoparticles effectively encapsulated siRNA (Figure 4c).

Prior to 3D tumor spheroid testing, the NP formulation was validated in 2D cell cultures of U87 MG cells. The results confirmed low cytotoxicity of PEI‐F and efficient cellular uptake, as well as gene silencing capability of the PEI‐F/siRNA/HA complex (Figures S6–S8). NP penetration was investigated by treating tumor spheroids with Cy5‐labeled siRNA‐loaded NPs (PEI‐F/siRNA‐Cy5/HA). Z‐stack images of spheroids, reaching depths up to 60 µm, were acquired using a confocal microscope (Figure 4d). Intense fluorescence signals were observed in the outer layers of the spheroids, with clear penetration into deeper regions. In contrast, treatment with free siRNA‐Cy5 (control) resulted in no detectable fluorescence signal, indicating that free siRNA was unable to penetrate the spheroid structure (Figure 4e). Quantitative analysis of Cy5 fluorescence intensity within MCF‐7 and U87 MG spheroids further demonstrated that NPs significantly enhanced siRNA delivery to the interior of the 3D tumor spheroids. Moreover, delivery efficiency was higher in U87 MG spheroids compared to MCF‐7 spheroids (Figure 4f).

### 2.5 Evaluation of Nanomedicine Therapy Using Tumor Spheroids

**FIGURE 5:**
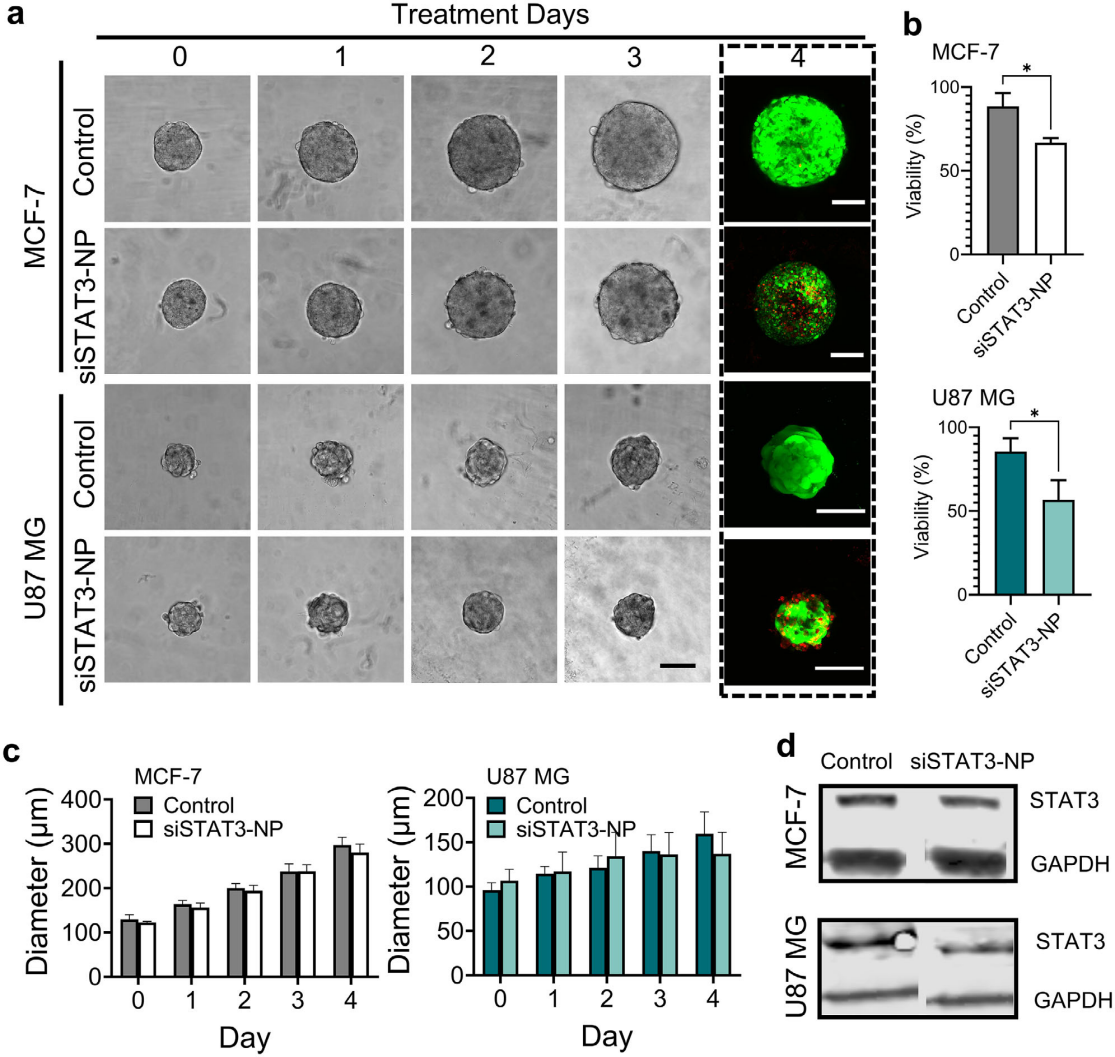
Evaluation of nanomedicine therapeutic effects using 3D tumor spheroids. (a) Representative brightfield images showing morphological changes of MCF‐7 and U87 MG spheroids following treatment with free siSTAT3 (control groups) or siSTAT3‐loaded nanoparticles (siSTAT3‐NP groups) on Day 0, 1, 2, and 3. Live/dead fluorescence images on Day 4 (highlighted in dotted boxes) show viability differences between treatment groups. Scale bars: 100 µm. (b) Quantitative analysis of cell viability of MCF‐7 and U87 MG spheroids after treatment (mean ± SD, *n* = 3). *p*‐values were calculated using a two‐tailed unpaired Student's *t*‐test. ^*^ *p* < 0.05, ^**^ *p* < 0.01, ^***^ *p* < 0.001, ^****^ *p* < 0.0001; NS, not significant. (c) Average diameter changes of MCF‐7 and U87 MG tumor spheroids over the treatment period in control and siSTAT3‐NP groups (mean ± SD, *n* = 3). (d) Representative western blot images showing STAT3 expression levels in spheroids from control and siSTAT3‐NP groups.

As a functional demonstration, the nanoparticle platform was used to deliver siRNA targeting the transcription factor STAT3 (signal transducer and activator of transcription 3), often overactivated in diverse human cancers. The siRNA‐loaded NPs can reach their target mRNA and mediate gene silencing, indicating potential therapeutic effects. However, limited penetration depth may compromise the delivery of therapeutic agents to the spheroid core, potentially reducing efficacy against inner tumor cell populations. We assessed the therapeutic effects of the siRNA‐based NPs using two distinct tumor spheroid models. Spheroid growth under nanomedicine treatment was monitored over a four‐day period, as shown in Figure 5a–c. Notably, siRNA‐loaded NPs targeting *STAT3* (siSTAT3‐NP) exhibited a more pronounced inhibitory effect in the U87 MG model compared to the MCF‐7 model. Live/dead cell assays conducted on Day 4 revealed a significantly higher proportion of dead cells in both groups treated with siSTAT3‐NP relative to control groups (treated with free siSTAT3).

In MCF‐7 spheroids, cell death was not restricted to the peripheral regions, despite nanoparticle penetration being initiated from the spheroid surface. This observation is consistent with the delayed nature of siRNA‐mediated gene silencing [37]. In addition, the compact architecture of MCF‐7 spheroids may promote metabolic stress in inner regions when peripheral cells are functionally inhibited [38]. In contrast, U87 MG spheroids exhibited more pronounced cell death at the periphery following siSTAT3‐NP treatment. Although siRNA‐mediated gene silencing is also inherently delayed in this model, the relatively loose spheroid architecture of U87 MG cells and the critical role of STAT3 signaling in glioblastoma cell survival may render peripheral cells more susceptible to gene silencing–induced loss of viability [39, 40]. Relatively higher CD44‐mediated nanoparticle uptake in U87 MG spheroids may further contribute to the peripheral loss of viability observed in this model [35, 41].

Western blot analysis of STAT3 protein expression levels revealed reduced STAT3 protein expression following siSTAT3‐NP treatment (Figure 5d). Quantitative analysis confirmed significantly reduced STAT3 expression in siSTAT3‐NP‐treated spheroids compared with free siRNA controls in both cell lines, with a greater reduction observed in U87 MG spheroids (Figure S9). This greater sensitivity of U87 MG cells to siSTAT3‐NP correlated with the more efficient nanoparticle penetration observed in Figure 4. To further explore the dose‐response behavior, tumor spheroids were treated with increasing concentrations of siSTAT3‐NPs. A clear concentration‐dependent reduction in spheroid viability was observed in both MCF‐7 and U87 MG models (Figure S10). These results demonstrate the utility of microdroplet‐generated 3D tumor spheroids for assessing nanotherapeutic efficacy, providing insights into both phenotypic outcomes (e.g., growth inhibition and cell viability) and molecular responses (e.g., target gene knockdown).

## 3 Discussion

To our knowledge, this study represents the first demonstration of utilizing spheroids generated via droplet microfluidics for high‐throughput evaluation of siRNA‐based nanotherapeutics. By leveraging the platform's ability to produce large quantities of uniform spheroids, the therapeutic efficacy of siRNA‐loaded nanoparticles was rapidly validated through quantitative analysis of protein expression. The uniformity, reproducibility, and scalability of the droplet‐based platform enhance the reliability of high‐throughput drug screening, highlighting its potential for the preclinical evaluation of novel therapeutics.

Other spheroid generation methods include low‐attachment plates, hanging drops [42, 43], microwells [44, 45], as well as alternative approaches such as microstructure‐based methods and hydrogel‐based 3D culture systems [46, 47, 48], which may have limitations in throughput, uniformity, or control over the local microenvironment. Compared with these approaches, the droplet microfluidic platform offers distinct advantages for siRNA nanotherapeutic evaluation. A key strength lies in its exceptionally high throughput, with spheroid production reaching the order of tens of thousands per hour [24]. In contrast, low‐attachment plates and hanging‐drop methods are limited to tens to hundreds of spheroids per experiment, while microwell‐based platforms typically generate only a few thousand spheroids due to spatial constraints [13]. This high‐throughput capability is particularly advantageous for siRNA‐based studies, as large numbers of spheroids are needed to obtain statistically reliable results and to mitigate the intrinsic variability of 3D tumor models [49]. Rapid generation of uniform spheroids enables efficient screening while reducing experimental time and batch‐to‐batch variability.

Another key advantage of droplet microfluidic spheroid generation is the precise control over initial cell encapsulation and the local microenvironment, which provides greater experimental flexibility [15]. In low‐attachment plates and hanging‐drop methods, spheroid formation and the local microenvironment largely depend on manual handling [50]. As a result, cells may settle unevenly, leading to heterogeneous spheroid structures. In microwell‐based platforms, spheroid growth is limited by the fixed geometry of the wells, and the open nature of the wells makes it difficult to independently control the microenvironment of individual spheroids [51]. This enhanced level of control facilitates the production of highly uniform spheroids and improves the reproducibility of nanoparticle penetration and gene silencing measurements. Furthermore, the ability to independently tune the microenvironment of each spheroid provides the flexibility to extend the system to more complex models in the future, such as spheroids encapsulated within an extracellular matrix for advanced therapeutic evaluations [52].

Consistent with previously reported droplet‐based spheroid generation platforms, our system maintains high‐throughput spheroid generation (50 000 droplets every 5 min) and narrow size distributions. In addition, the generated spheroids exhibit high viability and can be released from droplets for sustained culture over extended periods. Importantly, while numerous droplet microfluidic platforms have been reported for spheroid generation, most prior studies have primarily focused on spheroid formation control, microenvironmental modulation, or phenotypic and biological characterization, such as morphology, viability, secretion, or therapy response [15, 26, 53, 54]. In contrast, very few studies have extended droplet‐generated spheroids to systematic, quantitative functional evaluation of nanomedicines, particularly using protein‐level readouts of siRNA‐mediated gene silencing across large spheroid populations [27]. In this context, the present work demonstrates that microdroplet‐generated spheroids can support high‐throughput, protein‐level functional assessment of siRNA‐mediated gene therapy, expanding the application scope of droplet‐based spheroid platforms toward quantitative nanomedicine evaluation. Overall, the combination of high throughput, precise control, and experimental flexibility establishes droplet microfluidics as a powerful platform for both current siRNA nanomedicine evaluation and future development of more complex 3D tumor models.

Differences in siRNA nanoparticle penetration between MCF‐7 and U87 MG spheroids were observed. These differences are consistent with the intrinsic structural characteristics of spheroids formed by these two cell types. MCF‐7 cells exhibit epithelial‐like properties and typically form highly compact spheroids with strong cell‐cell adhesion, whereas U87 MG glioblastoma cells generate loose and less compact spheroids [40, 55]. Such differences in spheroid architecture are known to influence transport properties, with looser spheroid structures being more permissive to the penetration of macromolecules and nanoparticle‐sized agents [56]. Similar structure‐dependent penetration behaviors have been reported in previous 3D tumor spheroid studies [8].

In addition, differences in the expression of CD44, a receptor involved in nanoparticle binding and cellular uptake, may contribute to the observed variation in penetration efficiency [57]. U87 MG cells have been reported to express relatively high levels of CD44, while CD44 expression in MCF‐7 cells is more variable depending on culture conditions [41, 55, 58]. Such differences in receptor availability may influence nanoparticle‐cell interactions at the spheroid periphery, contributing to distinct penetration behaviors. Beyond cell line‐specific effects, our results suggest that nanomedicine efficacy within 3D tumor spheroids is closely linked to nanoparticle penetration, highlighting penetration optimization as a promising direction for improving therapeutic delivery. Strategies aimed at reducing structural compactness may further improve therapeutic accessibility. Future work should explore the broader applicability of this platform to additional classes of therapeutics (e.g., immunotherapies and CRISPR‐based agents).

Despite the promising results demonstrated, several challenges remain. First, the size of spheroids generated within droplets is currently constrained by the confined space and restricted nutrient availability. While releasing tumor spheroids from droplets allows for further growth and downstream applications, this compromises the advantage of maintaining isolated microenvironments for each tumor spheroid. Although fusing multiple small spheroids offers a theoretical path to generating larger spheroids, reliably producing large spheroids with consistent size and morphology remains technically challenging. Moreover, cell sedimentation over time at the inlet compromises the consistency of cell encapsulation, particularly during extended droplet generation periods. After release from microdroplets, high spheroid density increases the likelihood of undesired fusion events. These limitations highlight the need for improved engineering solutions, including enhanced droplet fusion control, improved cell suspension stability, and post‐release handling strategies that preserve spheroid individuality. Addressing these challenges will further enhance the platform's utility as a powerful, scalable, and physiologically relevant model for early‐stage drug discovery and mechanistic studies of therapeutic delivery.

## 4 Conclusion

In this study, we developed a droplet microfluidic system for the high‐throughput generation of uniform tumor spheroids and demonstrated its utility in evaluating nanoparticle‐based therapeutics. The system offers precise control over spheroid formation, enabling scalable and reproducible production with minimal size variability. Spheroids released from the droplets maintained high viability and demonstrated sustained growth in both liquid suspension and 3D matrix environments. Moreover, the therapeutic effects of siRNA‐loaded nanoparticles on tumor spheroids were effectively validated through quantitative protein expression analysis and viability assays. These results confirmed the platform's capability to facilitate the assessment of nanomedicine performance in a controlled, high‐throughput format. Overall, this work establishes a robust methodology for nanoparticle screening in 3D tumor models, offering a more accurate and scalable approach to preclinical drug evaluation. We anticipate that the platform has strong potential to inform rational therapeutic design and optimization, and provides a foundation for future developments in advanced drug screening technologies and personalized medicine strategies.

## 5 Methods

### 5.1 Microfluidic Device Fabrication

The device pattern was designed using AutoCAD 2022 software. The device included two inlets. One inlet was for the oil phase, and the other was for the aqueous phase containing cells. There was one outlet for collecting the droplets. The flow‐focusing junction for droplet formation has a channel width of 70 µm and a depth of 100 µm. The device pattern was transferred onto a silicon wafer (University Wafer) using a standard photolithography process. A Micro Writer ML 3 lithography system was used for the exposure process, which can write patterns on the SU‐8 2075 photoresist (SU‐8, Micro Materials) directly without a film mask. After developing the pattern and performing hard baking, the SU‐8 mold was treated with trichlorosilane (Sigma–Aldrich) for 15 min under a chemical fume hood to prevent polydimethylsiloxane (PDMS) adhesion. PDMS (Sylgard 184, 1:10 curing agent to base, Motion Australia) was mixed, degassed, and cast onto the mold. The mold was baked at 65°C overnight to allow complete curing of the PDMS. Patterned PDMS was demolded and punched (1.25 mm) for inlets and outlets. The device was then plasma‐treated and bonded to clean glass slides. Prior to droplet generation, the devices were hydrophobically treated by baking at 65°C for approximately three days.

### 5.2 Cell Culture

MCF‐7 cells were kindly provided by Dr. Wei Zhang from Macquarie University. U87 MG cells were obtained from the American Type Culture Collection. Both MCF‐7 and U87 MG cells were cultured in DMEM containing 10% fetal bovine serum (FBS) and 1% penicillin‐streptomycin (all from Thermo Fisher Scientific). Cells were passaged or used for experiments when they reached 80%–90% confluency.

### 5.3 Encapsulation of Tumor Cells in Droplets

All devices and accessories, including two pumps, tubing, and connectors, were sterilized under UV light for 30 min. In our system, PicoSurf 1 (2% w/w in Novec 7500, Capella Science) was used as the oil phase. For the aqueous phase, cells were detached from the flask using Trypsin‐EDTA (Thermo Fisher Scientific), centrifuged at 1000 rpm for 5 min, and resuspended in fresh medium to the desired concentration after counting with a TC20 Automated Cell Counter (Bio‐Rad). The droplet generation system was set up in three steps. First, a 4–5 cm section of tubing was used to connect the chip outlet to a sterilized 0.5 mL (or 1.5 mL) Eppendorf tube. Second, a 1 mL syringe filled with fluorinated oil was connected to one inlet of the chip. Lastly, the cell suspension was loaded into a syringe and connected to the other inlet. It is important to note that the cell suspension must be thoroughly mixed before loading to ensure uniformity. To initiate the process, the pump controlling the oil phase was activated first. Once the air bubbles within the microchannels were expelled, the pump for the aqueous phase was immediately started.

### 5.4 Release of Tumor Spheroids from Droplets

After approximately 21 h of incubation, the tumor spheroids generated in droplets were released for subsequent analysis and further culture. A physical method to release spheroids was as follows: first, a defined volume (e.g., 10 µL) of fluorinated oil was pipetted onto a clean petri dish. An equal volume of droplets containing the spheroids was carefully dispensed onto the oil surface in the dish. After waiting for approximately 3 min, all the droplets were broken, releasing the tumor spheroids. Subsequently, 10 µL of fresh medium was added to the sample area, where the spheroids spontaneously aggregated. Using a pre‐wetted pipette tip, the spheroids were collected into a low‐attachment dish for continued culture.

### 5.5 Live/Dead Assay

LIVE/DEAD Viability/Cytotoxicity Kit (Thermo Fisher Scientific) was used to evaluate the viability of samples. The staining solution was prepared by mixing Calcein‐AM (C‐AM) and ethidium homodimer‐1 (EthD‐1) in PBS (Thermo Fisher Scientific) at a ratio of 2.5 µL:10 µL:5 mL (Calcein‐AM: EthD‐1: PBS). The samples were incubated with staining solutions at 37°C for 30 min, then washed three times with cell culture medium before fluorescence imaging.

### 5.6 Immunofluorescence Staining for Spheroids

The spheroids were transferred to 1.5 mL Eppendorf tubes and allowed to settle for 3 min, after which the supernatant was carefully removed. The samples were fixed in 4% paraformaldehyde (PFA, Sigma–Aldrich) for 25 min and washed thoroughly with PBS. Permeabilization was conducted with 0.1% Triton X‐100 (Sigma–Aldrich) for 30 min and blocked with 3% bovine serum albumin (BSA, Sigma–Aldrich) in TBST (Thermo Fisher Scientific) for 1 h at room temperature. For immunostaining, spheroids were incubated overnight at 4°C with primary antibodies diluted in 3% BSA. The following day, spheroids were washed three times with TBST, followed by three washes with TBS (Thermo Fisher Scientific). A 5‐min settling period was allowed between each wash to ensure the spheroids settled at the bottom of the tube. Next, the samples were incubated with secondary antibodies for 2 h at room temperature. After additional TBST and TBS washes, Hoechst 33342 (Thermo Fisher Scientific) and Cell Mask Orange Actin Tracking Stain (Thermo Fisher Scientific) diluted in PBS (1:1000 dilution) were added to stain the samples. Finally, the spheroids were washed thoroughly with PBS (eight times, 5 min each) and imaged using Olympus FV3000RS confocal microscope. A list of primary and secondary antibodies is provided in Tables S1 and S2.

### 5.7 Spheroid Invasion in 3D Gel

The collagen solution with a final concentration of 4 mg/mL was prepared using 3D Culture Matrix Rat Collagen I (R&D Systems), NaHCO_3_ (Sigma–Aldrich), and HEPES (Gibco) on ice. Approximately 5 spheroids were mixed into 100 µL of the collagen solution, which was then dropped into a 24‐well plate. The well plate was then incubated upside down for 15 min to allow the collagen solution to polymerize. Subsequently, the samples were cultured in the cell culture medium. Spheroid growth was monitored using the confocal microscope at 0, 4, 8, 24, 48, 72, and 96 h.

### 5.8 Preparation of siRNA‐Loaded Nanoparticles

We used a reported experimental method to prepare siRNA‐loaded nanoparticles [59, 60]. Branched polyethyleneimine (PEI, 1.8K, Thermo Fisher Scientific) was dissolved in methanol at 40 mg/mL and maintained in an ice bath under magnetic stirring. After 30 min, 200 µL of anhydrous triethylamine was added to the PEI solution. Subsequently, 220 µL heptafluorobutyric anhydride (Sigma–Aldrich) in 10 mL methanol was added dropwise (3 drops per second) to the mixture using a constant‐pressure dropping funnel. The reaction mixture was stirred for 48 h and then dialyzed against double‐distilled water for 3 days. The resulting product, designated as PEI‐F, was lyophilized into a white powder for future use.

For siRNA loading, 10 µL of siRNA (100 µm) was dispersed in 50 µL of HEPES buffer (10 mm, pH 7.4). Separately, 10 µL of PEI‐F (10 mg/mL) was dissolved in 50 µL of HEPES buffer. The siRNA solution was then added to the PEI‐F solution and allowed to incubate for 10 min to facilitate the formation of PEI‐F/siRNA complexes. Subsequently, 40 µL of sodium hyaluronate (35 kDa, 10 mg/mL) was added to the PEI‐F/siRNA solution to form PEI‐F/siRNA/HA nanoparticles. The size and zeta potential of the prepared PEI‐F/siRNA/HA nanoparticles were measured by dynamic light scattering (DLS) instrument (Malvern Zetasizer), and the morphology of the nanoparticles was imaged with TEM. The resulting nanoparticles were then stored at 4°C for further use. The sequences used in this study were as follows: STAT3 siRNA: sense 5’‐GGA CGA CUU UGA UUU CAA Ctt‐3’, antisense 5’‐GUU GAA AUC AAA GUC GUC Ctg‐3’; Scrambled siRNA: sense 5’‐UUC UCC GAA CGU GUC ACG UdTdT‐3’, antisense 5’‐ACG UGA CAC GUU CGG AGA AdTdT‐3’. Cy5‐labeled scrambled siRNA was used for imaging. All the siRNA were obtained from GenePharma (Shanghai, China).

### 5.9 Evaluation of siRNA‐Load Nanomedicine in 2D Cell Cultures

For the cytotoxicity assay, a total of 5000 U87 MG cells were seeded into each well of a 96‐well plate and maintained overnight in a CO_2_ incubator. The culture medium was exchanged for fresh medium containing different concentrations of PEI‐F/siRNA and PEI25K/siRNA. After 24 h of incubation, cell viability was evaluated using the CCK‐8 assay.

For cellular uptake analysis, U87 MG cells (2 × 10^5^ cells per well) were seeded into 12‐well plates containing cover slides. After 24 h of cell culture, U87 MG cells were incubated in fresh culture medium supplemented with free siRNA‐Cy5 and PEI‐F/siRNA‐Cy5/HA at a final siRNA concentration of 400 nm and incubated for 3 h at 37°C. The treated samples were fixed and stained with DAPI as previously described. Imaging was performed using a Zeiss LSM 880 laser‐scanning confocal microscope.

For western blot (WB) analysis, U87 MG cells were seeded at 2 × 10^5^ cells per well in 12‐well plates and cultured for 24 h. The cells were then exposed to culture medium containing different nanoparticle formulations (Figure S7) for 48 h. After treatment, cells were washed and collected for protein extraction. WB was used to quantitatively evaluate the silencing efficiency of the siSTAT3 gene. In the WB analysis, the extracted proteins from all groups were separated by 4∼15 % sodium dodecyl sulfate‐polyacrylamide gel electrophoresis (SDS‐PAGE) and transferred to a nitrocellulose membrane. The membranes were blocked in 5% BSA for 1 h and incubated with anti‐STAT3 primary antibody (Abcam) and anti‐GAPDH primary antibody (Proteintech) at 4°C overnight, followed by washing with TBST twice (5 min each) and TBS twice (5 min each). Subsequently, the washed membranes were incubated with secondary antibodies (Licor) in 5% BSA for 1 h at room temperature. After incubation, the membranes were washed with TBST and TBS, and the protein bands were visualized using a Licor Odyssey Clx system. Antibody details are provided in Tables S1 and S2.

### 5.10 Evaluation of siRNA‐Load Nanomedicine in 3D Tumor Spheroids

During the release of tumor spheroids, the released spheroids from 10 µL of droplets were transferred into each well of a low‐attachment 6‐well plate for further culture. Two wells were prepared for each spheroid type (U87 MG and MCF‐7), with one serving as the experimental group and the other as the control. On Day 3, PEI‐F/siRNA/HA nanodrug was added to two experimental wells at a final siRNA concentration of 0.2 µm. For the control group, free siSTAT3 was added to the remaining two wells at the same final concentration of 0.2 µm. After four days of treatment, the samples were collected from the low‐attachment 6‐well plate for subsequent WB experiments as previously described. For morphology tracking, five spheroids were individually selected from each well and transferred to a low‐attachment 96‐well plate for further culture. The morphological changes were observed daily.

### 5.11 Statistical Analysis

Statistical analysis was performed using GraphPad Prism 10 (version 10.4.1). Data were presented as mean ± standard deviation (SD), unless otherwise stated in the corresponding figure legends. No data transformation or normalization was applied unless explicitly specified. For comparisons between two groups, a two‐tailed unpaired Student's *t*‐test was used. For comparisons involving more than two groups, two‐way analysis of variance (ANOVA) followed by Sidak's multiple comparisons test was performed. Differences were considered statistically significant at *p* < 0.05. For each condition, spheroids were randomly selected for quantitative analysis. The number of biological replicates (*n*) for each experiment is indicated in the corresponding figure legends. Experiments affected by accidental sample mixing or technical handling errors were excluded from analysis prior to statistical evaluation.

## Conflicts of Interest

The authors declare no conflicts of interest.

## Supporting information




**Supporting File**: adhm70972‐sup‐0001‐SuppMat.docx

## Data Availability

The data that support the findings of this study are available from the corresponding author upon reasonable request.
