# Supplementary material for: High‐Throughput Generation of Tumor Spheroids via Droplet Microfluidics for siRNA‐Loaded Nanomedicine Assessment
Source: Adv Healthc Mater. 2026 Feb 21;15(17):e03604. doi: 10.1002/adhm.202503604 (PMC13175295; doi:10.1002/adhm.202503604)
Supplement: Supplementary file 1 — Supporting File: adhm70972‐sup‐0001‐SuppMat.docx [file ADHM-15-0-s001.docx]

High-throughput Generation of Tumor Spheroids via Droplet Microfluidics for siRNA-Loaded Nanomedicine Assessment

Ling Liu^a,d^, Guoying Wang^b,^ * Yang Zhang^d^, Bingyang Shi^c^, and Ming Li^a, d,^ *

*a. School of Engineering, Macquarie University, Sydney, NSW 2109, Australia*

*b. Macquarie Medical School, Faculty of Medicine, Health and Human Sciences, Macquarie University, Sydney, New South Wales 2109, Australia*

*c. School of Biomedical Engineering, The University of Technology Sydney, Sydney, NSW 2007, Australia*

*d. School of Mechanical and Manufacturing Engineering, The University of New South Wales, Sydney, NSW, 2052 Australia*

E-mail: [guoying.wang@mq.edu.au](mailto:guoying.wang@mq.edu.au); [ming.li3@unsw.edu.au](mailto:ming.li3@unsw.edu.au)

**Table S1** Primary antibodies used in immunofluorescence and Western blotting

| Protein | Species | Vendor | Catalog number | Ratio |
| --- | --- | --- | --- | --- |
| GFAP | Rabbit | Thermo Fisher Scientific | PA1-10019 | 1: 500 |
| ERα | Rabbit | Sigma–Aldrich | 06-935 | 1: 500 |
| STAT3 | Rabbit | Abcam | EPR787Y | 1:1000 |
| GAPDH | Mouse | Proteintech | 60004-1-Ig | 1:10000 |

**Table S2** Secondary antibodies used in immunofluorescence and Western blotting

| Antibody | Wavelength | Vendor | Catalog number | Ratio |
| --- | --- | --- | --- | --- |
| Goat anti-Rabbit  Alexa Fluor 488 | 488 | Thermo Fisher Scientific | A32723 | 1：300 |
| Goat anti-Rabbit  Alexa Fluor 594 | 594 | Thermo Fisher Scientific | A32740 | 1：300 |
| IRDye® 680RD Donkey anti-Mouse IgG Secondary Antibody | 680 | Licor | 926-68072 | 1:10000 |
| IRDye® 800CW Donkey anti-Rabbit IgG Secondary Antibody | 800 | Licor | 926-32213 | 1:10000 |

**
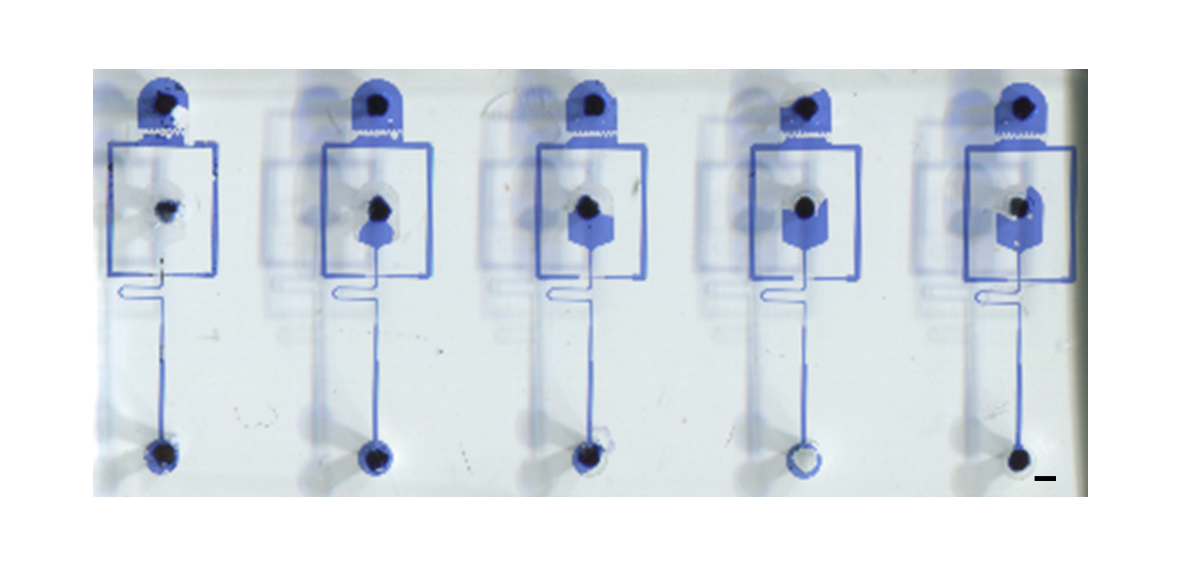
**

**Figure S1.** A representative image of the droplet-based microfluidic chips used in this study. Scale bar: 1 mm.

**
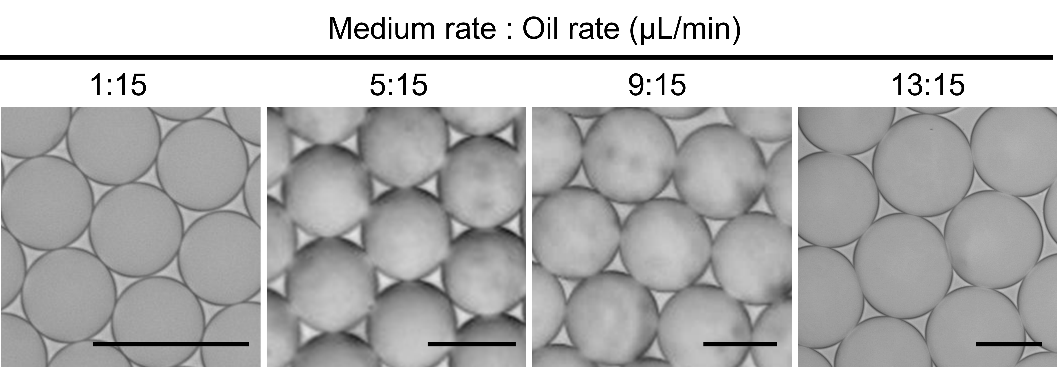
**

**Figure S2.** Representative images showing changes in droplet diameter under different flow conditions at a fixed oil flow rate of 15 μL/min. Scale bars: 100 μm.


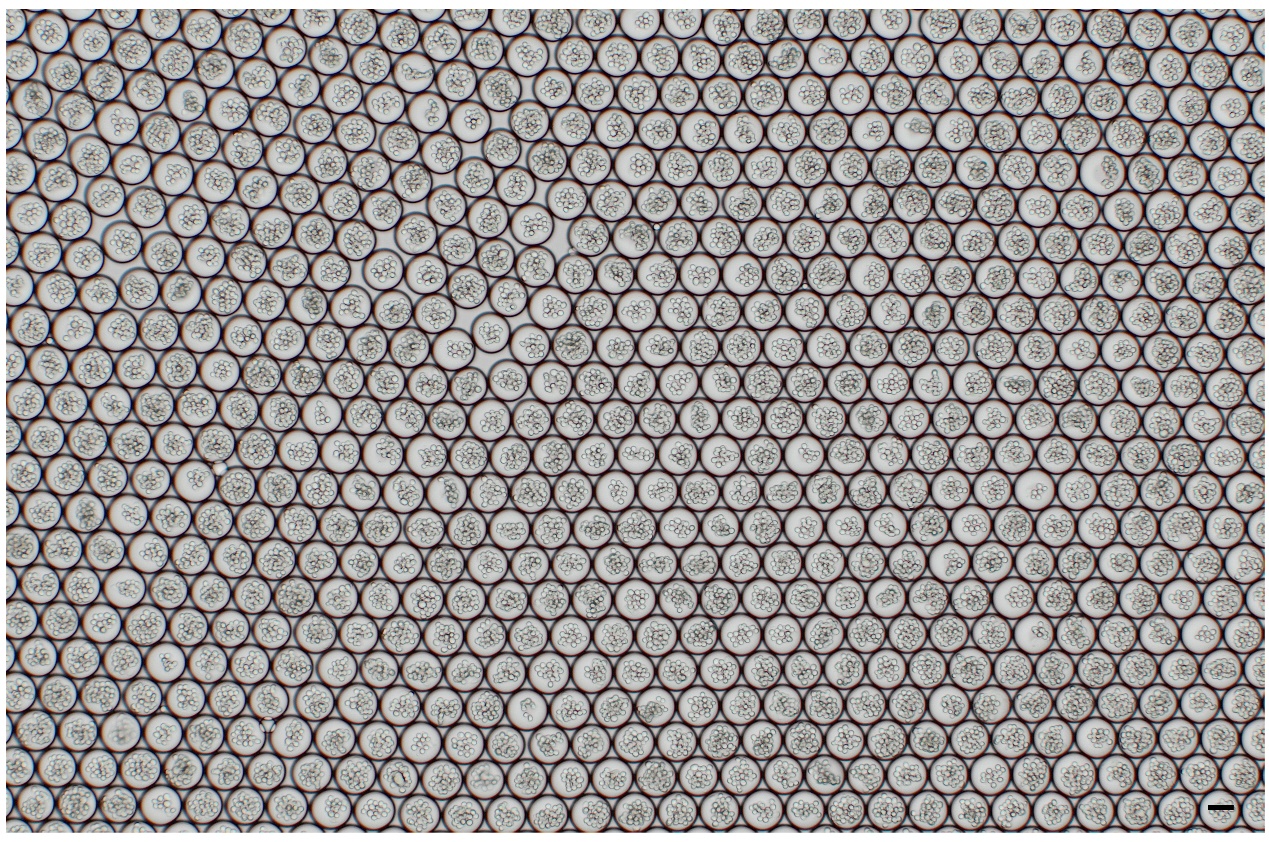


**Figure S3.** A representative image of tumor cells encapsulated in microdroplets generated at a water-to-oil flow ratio of 30:50 μL/min. The cell concentration is 40 million/mL. Scale bar: 100 μm.


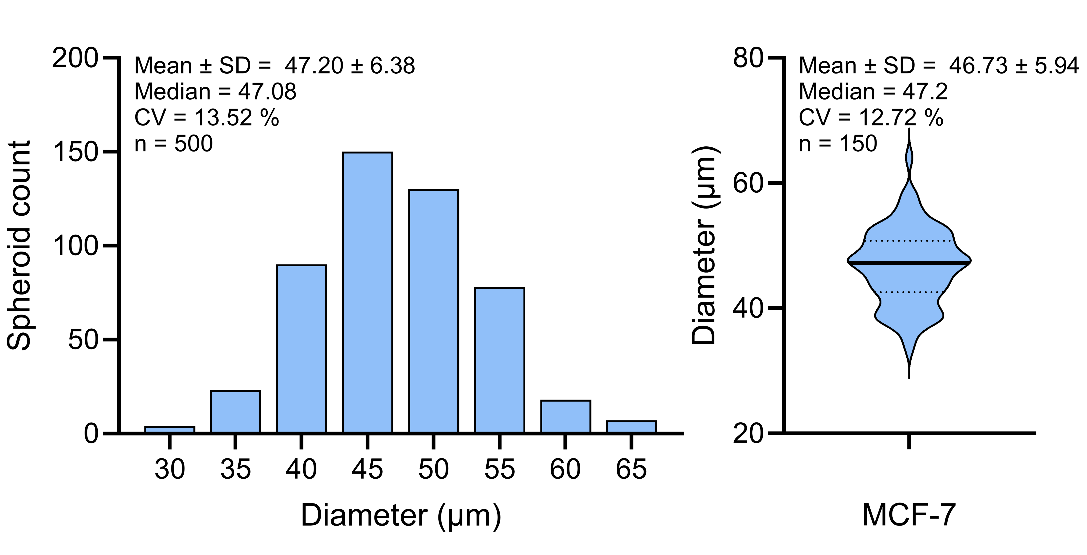


**Figure S4.** Size distribution and reproducibility of spheroids generated by the droplet microfluidic platform. The histogram shows the diameter distribution of spheroids generated within a single representative batch, illustrating size homogeneity (n = 500 spheroids). The violin plot displays pooled spheroid diameters from three independent batches (n = 150 spheroids), demonstrating the overall size distribution and consistent size control of the platform across experiments. The solid horizontal line indicates the median, while the dashed horizontal lines represent the first and third quartiles (25th and 75th percentiles).


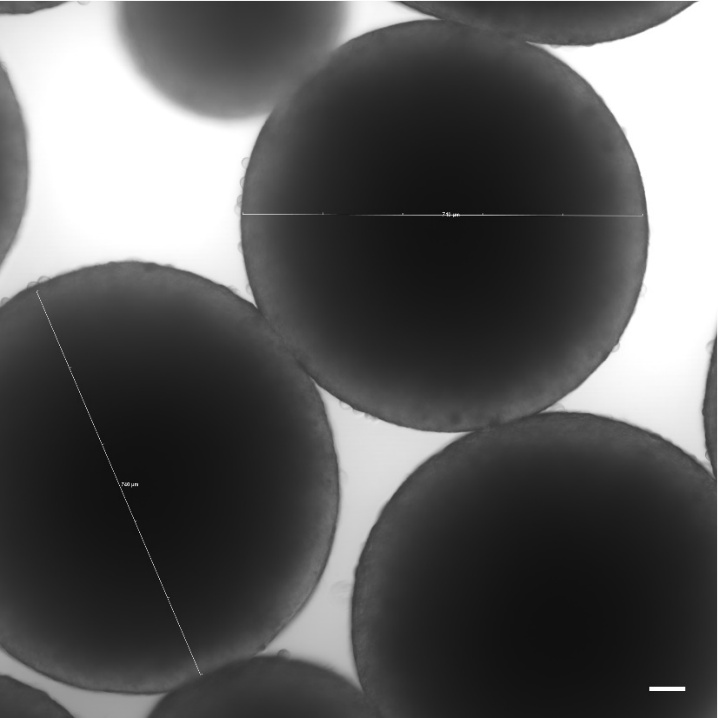


**Figure S5.** A representative image showing the formation of a large spheroid (≥700 μm in diameter) within 7 days of culture, resulting from the fusion of smaller spheroids. Scale bar: 100 μm.


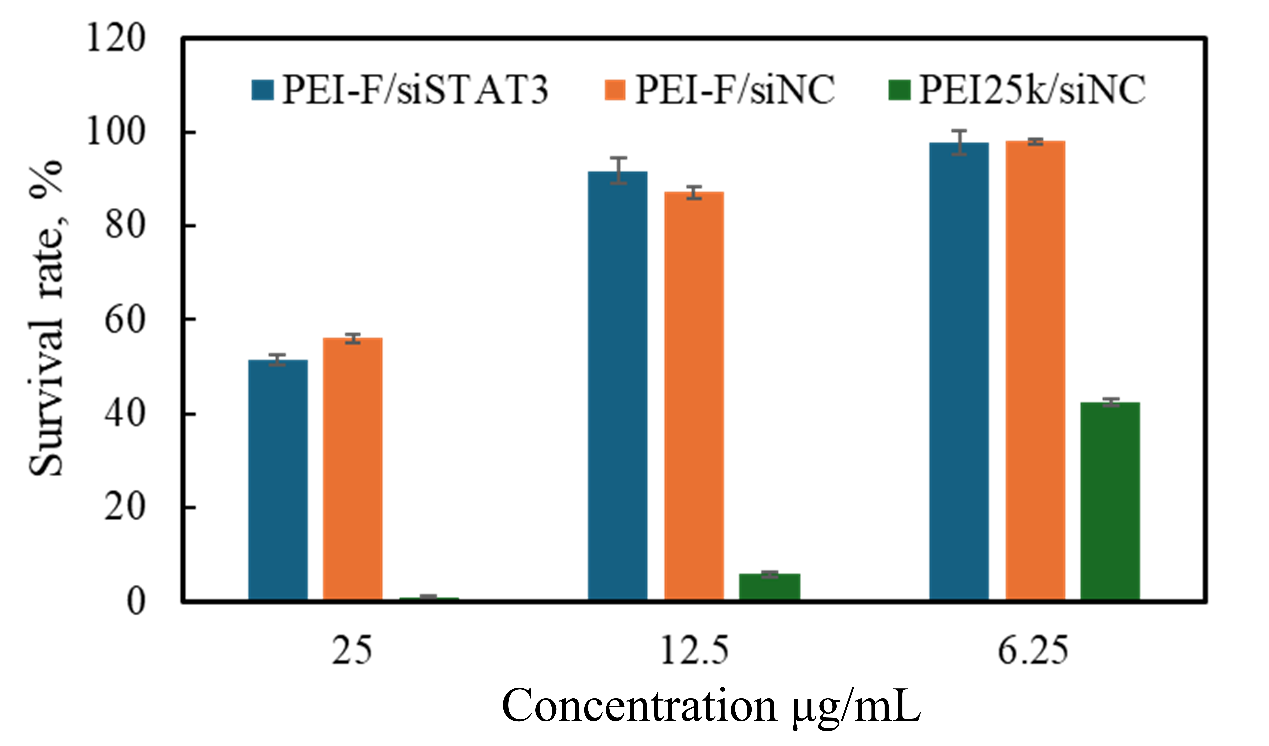


**Figure S6.** Cytotoxicity analysis in U87 MG cells after treatment with various nanoparticle formulations, with the concentrations of PEI-F or PEI25K at 25, 12.5 and 6.25 μg/mL. The weight ratio between PEI-F or PEI25K and siRNA was set at 10:1. (mean ± SD, n = 3).


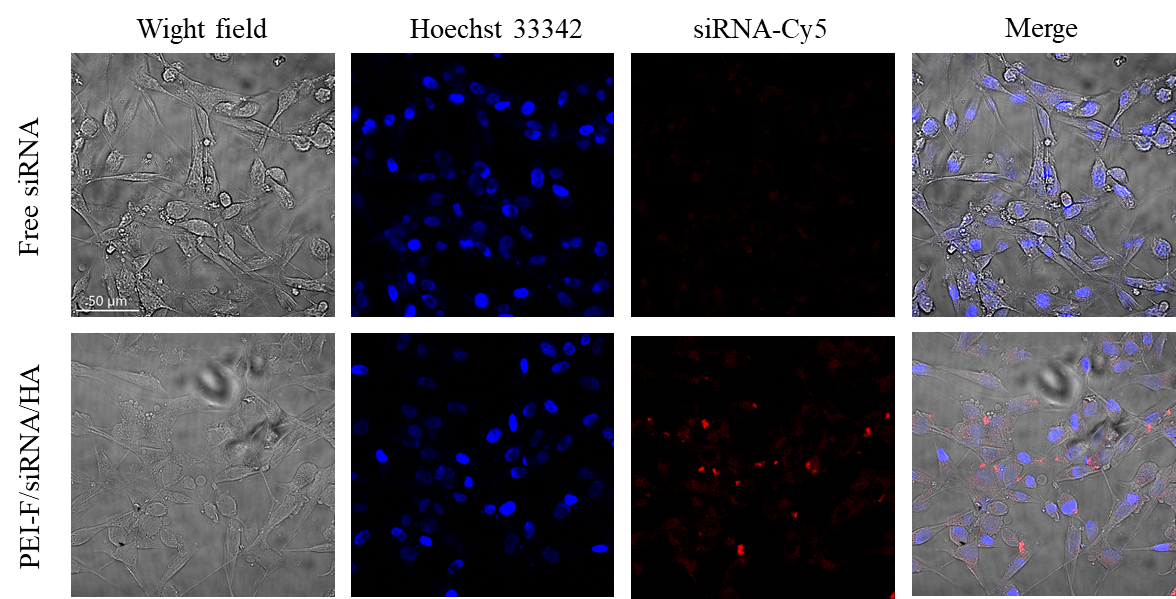


**Figure S7.** Confocal microscopy images showing the cellular uptake of free siRNA and PEI-F/siRNA/HA nanoparticles in U87 MG cells. Images were captured after 3 hours of incubation. Scale bar: 50 μm.


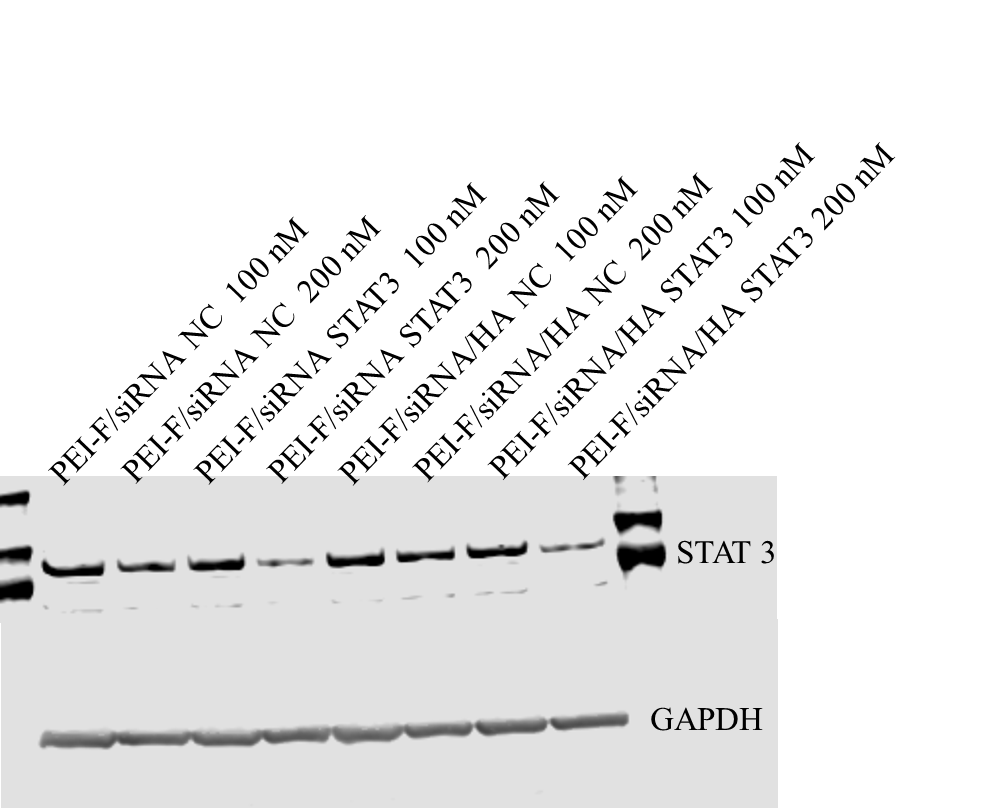


**Figure S8.** Western blot analysis of STAT3 protein expression in U87 MG cells following treatment with various nanoparticle formulations. PEI-F-based nanoparticles effectively reduced target protein expression (42.3±4.2%), while the HA-modified nanoparticles showed higher gene silencing efficiency (53.0±8.9%). These results demonstrated the superior delivery and gene silencing capability of the PEI-F-HA-functionalized nanoparticle system, based on three independent experiments (n = 3).


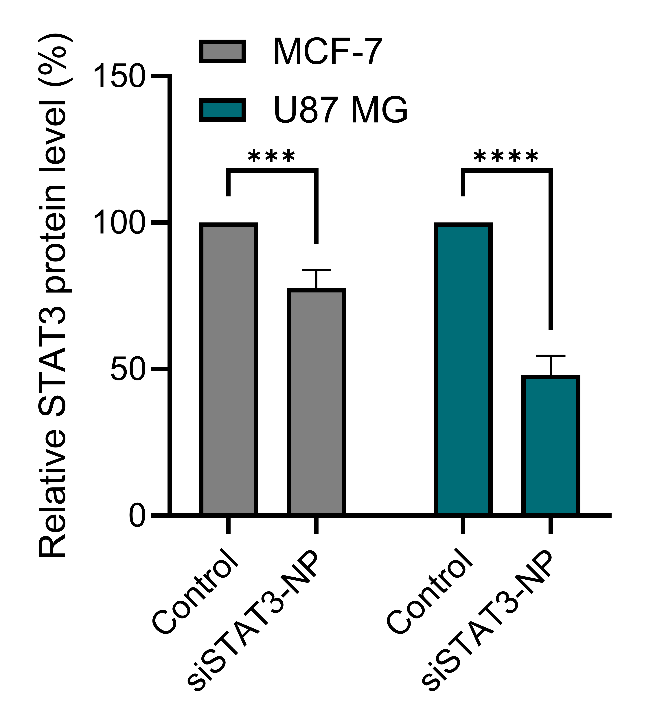


**Figure S9.** Quantitative analysis of STAT3 protein expression in tumor spheroids following nanomedicine treatment. Western blot band intensities corresponding to STAT3 expression in MCF-7 and U87 MG spheroids treated with free siSTAT3 (control) or siSTAT3-loaded nanoparticles (siSTAT3-NP) were quantified using Image Studio 6.1 and normalized to the corresponding loading control. Data are presented as mean ± SD (n = 3 independent experiments). Statistical significance was determined using two-way ANOVA followed by Sidak’s multiple comparisons test. *p < 0.05, **p < 0.01, ***p < 0.001, ****p < 0.0001; NS, not significant.


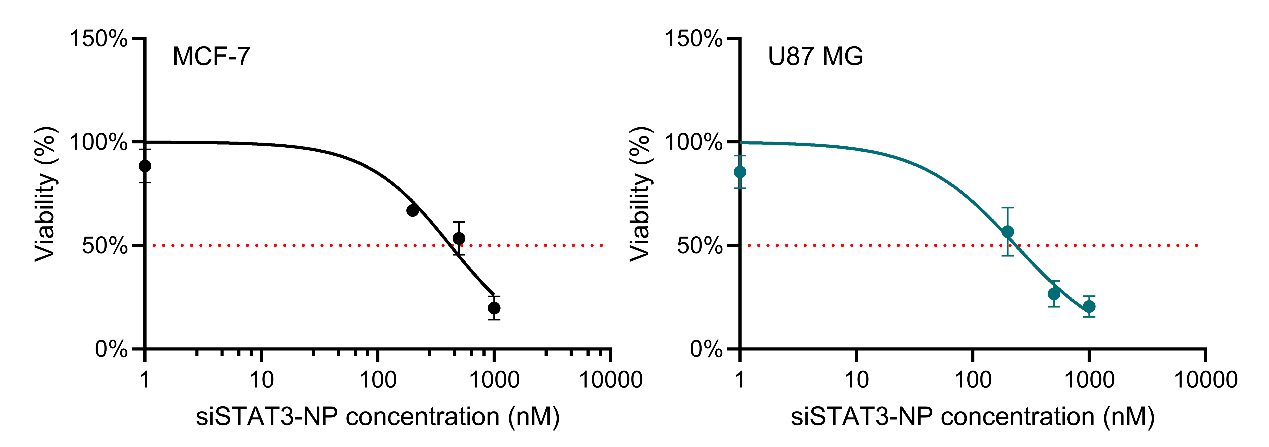


**Figure S10.** Dose-response curves of siSTAT3-NPs in MCF-7 and U87 MG tumor spheroids. Dose-response curves were fitted using nonlinear regression to illustrate concentration-dependent trends. Data are presented as mean ± SD (n = 3).
